# Pancreatic Cancer and Cellular Senescence: Tumor Microenvironment under the Spotlight

**DOI:** 10.3390/ijms23010254

**Published:** 2021-12-27

**Authors:** Michela Cortesi, Michele Zanoni, Francesca Pirini, Maria Maddalena Tumedei, Sara Ravaioli, Ilario Giovanni Rapposelli, Giovanni Luca Frassineti, Sara Bravaccini

**Affiliations:** 1 Biosciences Laboratory, IRCCS Istituto Romagnolo per lo Studio dei Tumori (IRST) “Dino Amadori”, 47014 Meldola, Italy; michele.zanoni@irst.emr.it (M.Z.); francesca.pirini@irst.emr.it (F.P.); maria.tumedei@irst.emr.it (M.M.T.); sara.ravaioli@irst.emr.it (S.R.); sara.bravaccini@irst.emr.it (S.B.); 2 Department of Medical Oncology, IRCCS Istituto Romagnolo per lo Studio dei Tumori (IRST) “Dino Amadori”, 47014 Meldola, Italy; ilario.rapposelli@irst.emr.it (I.G.R.); luca.frassineti@irst.emr.it (G.L.F.)

**Keywords:** senescence, pancreatic cancer, tumor microenvironment, SASP, senotherapeutics

## Abstract

Pancreatic ductal adenocarcinoma (PDAC) has one of the most dismal prognoses of all cancers due to its late manifestation and resistance to current therapies. Accumulating evidence has suggested that the malignant behavior of this cancer is mainly influenced by the associated strongly immunosuppressive, desmoplastic microenvironment and by the relatively low mutational burden. PDAC develops and progresses through a multi-step process. Early in tumorigenesis, cancer cells must evade the effects of cellular senescence, which slows proliferation and promotes the immune-mediated elimination of pre-malignant cells. The role of senescence as a tumor suppressor has been well-established; however, recent evidence has revealed novel pro-tumorigenic paracrine functions of senescent cells towards their microenvironment. Understanding the interactions between tumors and their microenvironment is a growing research field, with evidence having been provided that non-tumoral cells composing the tumor microenvironment (TME) influence tumor proliferation, metabolism, cell death, and therapeutic resistance. Simultaneously, cancer cells shape a tumor-supportive and immunosuppressive environment, influencing both non-tumoral neighboring and distant cells. The overall intention of this review is to provide an overview of the interplay that occurs between senescent and non-senescent cell types and to describe how such interplay may have an impact on PDAC progression. Specifically, the effects and the molecular changes occurring in non-cancerous cells during senescence, and how these may contribute to a tumor-permissive microenvironment, will be discussed. Finally, senescence targeting strategies will be briefly introduced, highlighting their potential in the treatment of PDAC.

## 1. Introduction

Despite therapeutic advances and the improvement of medical technologies, pancreatic cancer remains a highly lethal cancer, representing the fourth leading cause of cancer death in both men and women [1]. Only approximately 20% of patients diagnosed with pancreatic ductal adenocarcinoma (PDAC) are eligible for treatment with curative intent (i.e., surgical resection in combination with adjuvant and/or pre-operative therapy) [2]. However, despite the complete resection of the tumor, the majority of patients will recur locally or at distant sites and die within 5 years [3]. PDAC’s negative prognosis is mainly ascribable to its late diagnosis, and great efforts have been made in recent years for the identification of early diagnosis biomarkers [4]. As compared to other solid tumors, PDAC shows great genetic intra- and inter-tumoral heterogeneity, contributing to a lack of clinical and molecular classification systems which are able to improve patient management [5]. Moreover, the abundant presence of dense stroma, together with tissue inflammation, makes this tumor unique [6]. The stroma surrounding PDAC may represent up to 90% of the whole tumor and is characterized by a low oxygen concentration, poor vascularization, and extensive fibrosis associated with variable immune infiltrate [7]. The fibrotic portion of the tumor consists of both cellular and non-cellular components, the former being mainly composed of cancer-associated fibroblasts (CAFs), endothelial cells, and pancreatic stellate cells (PSCs) while the latter is composed of different extracellular matrix (ECM) molecules, such as collagen, glycosaminoglycans, proteoglycans, and growth factors, further enhancing tumor heterogeneity [8].

Senescence is a well-known tumor suppressor mechanism; however, paracrine signaling of senescent cells has recently gained attention for its pro-tumorigenic effect. Different stimuli are known to trigger cellular senescence, in particular those inducing genotoxic, oncogenic, oxidative, and replicative stress [9,10]. Regardless of the stimuli, senescent cells share some common behaviors, among which the main ones are growth arrest, apoptosis resistance, sustained DNA damage signaling, and heterochromatin modifications [11]. Despite an inability to proliferate, these cells still remain metabolically active and secrete a plethora of molecules—termed, as a whole, as senescent-associated secretory phenotype (SASP)—fostering cellular senescence through both autocrine and paracrine signaling [12,13,14]. Importantly, the SASP is considered the main driver of senescence-related pro-tumorigenic effects, as it can be involved in cancer relapse and metastases, transforming the neighboring cells (both tumor and non-tumor), and destructing the ECM [15,16,17].

In this review, the hallmarks and the mechanisms of senescence induction will be summarized, and the role of senescence in PDAC progression with respect to the heterogeneity of the tumor will be addressed. Specifically, the senescence-related cellular and molecular changes occurring in the TME and how they may contribute to a tumor-permissive microenvironment will be discussed. Finally, future therapeutic options targeting senescent cells will be briefly introduced and their potential discussed in the treatment of PDAC.

## 2. Pancreatic Ductal Adenocarcinoma Heterogeneity

### 2.1. Genetic Heterogeneity

**Figure 1:**
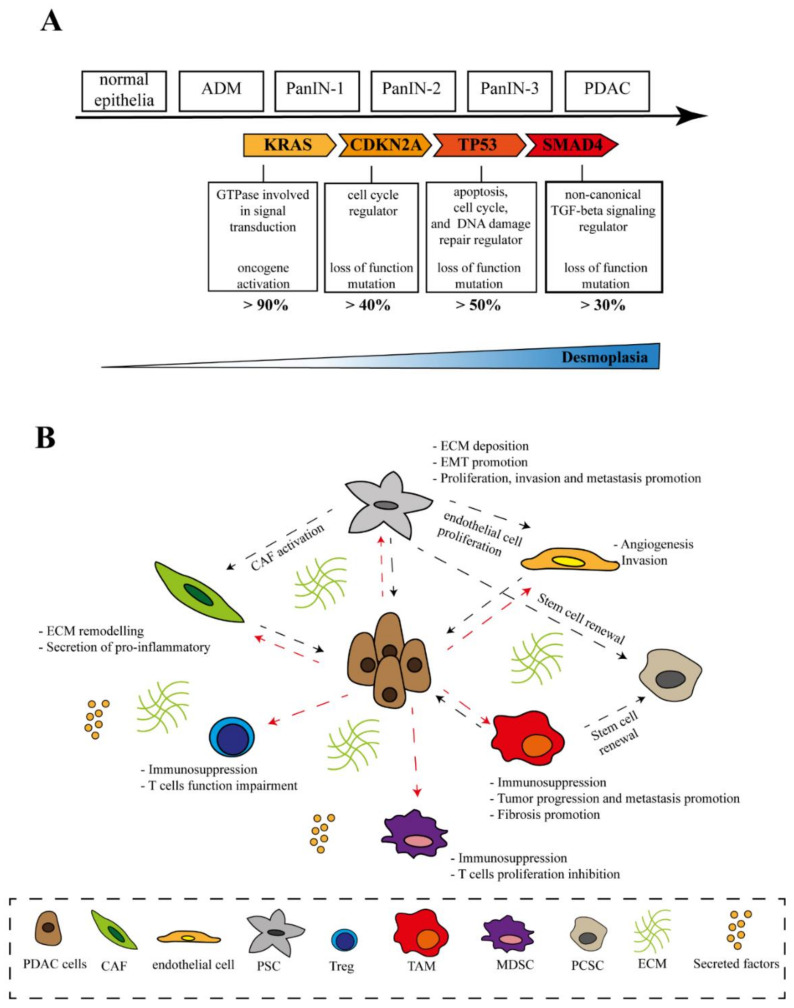
Pancreatic cancer heterogeneity. (**A**) The progression model of PDAC is driven by KRAS, TP53, CDKN2A, and SMAD4 mutations, which modulate tumor initiation and progression; (**B**) cross-talk between tumor cells and non-tumor cells in PDAC microenvironment. Abbreviations: PDAC, pancreatic ductal adenocarcinoma; CAF, cancer-associated fibroblast; PSC, pancreatic stellate cell; Treg, regulatory T cell; TAM, tumor-associated macrophage; MDSC, myeloid-derived suppressor cell; PCSC, pancreatic cancer stem cell; ECM, extracellular matrix.

PDAC is the most common type of pancreatic cancer, arising from the exocrine compartment of the pancreas. The actual progression model of PDAC describes the transformation of acinar cells into ductal-like cells as the initial step of tumorigenesis as a consequence of a stressful event (i.e., tissue damage, inflammation). This transformation makes the cells more susceptible to pro-oncogenic events, thus promoting the onset of intra-epithelial neoplasias (PanINs), followed by sequential tumor suppressor gene mutations, leading to cancer progression [18]. It has been well-established that four main mutational events progressively accumulate in PDAC, driving the tumorigenesis: Kirsten rat sarcoma viral oncogene homolog (KRAS) activation, followed by cyclin-dependent kinase inhibitor 2A (CDKN2A), tumor protein p53 gene (TP53), and SMAD family member 4 (SMAD4) inactivation [5,19,20]. KRAS mutations show the highest frequency in PDAC, with more than 90% of tumors harboring oncogenic point mutations of this gene. These mutations are considered a marker of poor prognosis and lead to a wide range of functional consequences [21]. Inactivating mutations of TP53 have been reported in more than 50% of pancreatic cancers and commonly cause apoptosis evasion, cell cycle progression, and disabling DNA damage repair [22]. Mutations in CDKN2A (>40%) are mainly related to cell cycle dysregulation, promoting cell cycle progression through the loss of cyclin-dependent kinase (CDK) 4 and CDK6 inhibition. Finally, more than 30% of PDAC have been reported to show SMAD4 mutations, which occur at the latest stages of tumorigenesis and promote cell survival through non-canonical TGF-beta signaling [23] (Figure 1A). In addition to these common alterations, a wide number of less-frequent mutations may occur (e.g., mutations in DNA damage repair genes [24], mismatch repair genes [25], PI3K/AKT/mTOR signaling pathways genes [26], and so on), resulting in genetic heterogeneity of PDAC [27]. Notably, such heterogeneity is not only due to genetic cues, but also due to epigenetic mechanisms which are capable of regulating both tumor suppressors and oncogenes [28]. Epigenetic regulatory genes, such as those encoding for chromatin remodeling complexes, histone methylases, histone methyltransferase, and histone demethylases, are indeed frequently mutated in PDAC [24,29]. The heterogeneous genetic nature of PDAC highlights the necessity of personalized treatment strategies based on patient stratification; however, a consensus classification is still lacking.

### 2.2. Tumor Microenvironment

Pancreatic cancer resides in an extensive and complex fibrous stroma consisting of a small, but heterogeneous, cellular fraction and a non-cellular fraction [30]. The so-called desmoplastic reaction of the tumor is a hallmark of PDAC, which is present in both primary and metastatic lesions and which makes this tumor unique when compared to other solid tumors [31]. The stromal reaction arises from pancreatic stellate cells (PSCs) that, once activated by tumor cells, start to deposit the fibrotic matrix [32]. In addition to PSCs, fibroblasts, immune cells, endothelial cells, and pancreatic cancer stem cells have been mainly identified in the TME [33] (Figure 1B). The cellular and non-cellular components of the TME maintain tissue homeostasis. The complex interplay between the TME and tumor cells can alternatively promote or suppress cancer development and progression based on context-dependent stromal alterations [34].

#### 2.2.1. Pancreatic Stellate Cells (PSCs)

PSCs are star-shaped quiescent stromal cells that, in their inactive state, serve as storage for vitamin A droplets, in addition to being involved in normal pancreas secretions, immunity, and in the maintenance of stroma homeostasis [35]. PSCs become activated once exposed to environmental factors—namely, oxygen variations and reactive oxygen species (ROS) generation—or to secretory molecules, such as cytokines, growth factors, and other signaling molecules [36]. Activated PSCs acquire proliferative and migratory capabilities, while showing an increased production of cytokines (e.g., IL-1, -6, -8, and -10) and growth factors (e.g., insulin-like growth factor 1 (IGF1); vascular endothelial growth factor (VEGF); platelet-derived growth factor (PDGF); fibroblast growth factor (FGF); and CXC motif chemokine 12 (CXCL12)) [37]. The production of signaling molecules sustains, through an autocrine loop, the activation of PSCs themselves and promotes the angiogenesis, proliferation, migration, and invasion of cancer cells through paracrine signaling [38]. However, the activation of these cells is not permanent, as PSCs can regress to a quiescent state under the influence of several processes, such as senescence or tissue recovery [38,39]. Interestingly, recent studies have shown that PSCs and cancer cells establish reciprocal interactions, which significantly contribute to PDAC growth and aggressiveness [40,41]. It has been reported that, the secretion of cytokines, such as IL-6, by PSCs seems to be involved in promoting the immunosuppressive properties of the TME and in supporting the invasiveness of PDAC [42]. The main marker of PSC activation is the expression of alpha-smooth muscle actin (α-SMA) together with the deposition of a high amount of extracellular matrix (ECM) [36]. The deposition of ECM molecules, such as collagens, hyaluronic acid, laminin, tenascin-C, and fibronectin, modulate the viability, adhesion, and migration properties of tumor cells [43]. This matrix also acts as a physical barrier, contributing to the intra-tumoral hypoxia, compressing blood vessels, and impairing drug delivery to PDAC cells [44]. Activated PSCs have been shown to be able to remodel the ECM in PDAC, promoting an increase in tissue stiffening and in the crosslinking of collagen fibers [45]. Moreover, they can also promote the epithelial–mesenchymal transition (EMT) of tumor cells, which fosters tumor invasion and metastasization. Indeed, it has been demonstrated that cancer cells located at the invasive front of the tumor show a decreased expression of epithelial markers (e.g., e-cadherin) and an increased expression of mesenchymal markers (e.g., vimentin) as a consequence of exposure to stromal cell signals [46].

#### 2.2.2. Cancer-Associated Fibroblasts (CAFs)

CAFs are one of the most represented and dynamic elements composing the PDAC stroma [47]. They may derive from different types of cells, including resident fibroblasts, bone marrow-derived mesenchymal stem cells, and epithelial cells; however, it is commonly believed that one of the main CAF sources in PDAC is activated PSCs [36,48,49]. CAFs and PDAC cells mutually promote each other’s proliferation and differentiation [50]. Importantly, CAF activation is dependent on a plethora of complex mechanisms involving pro-inflammatory signals [51], changes in physical ECM properties [52,53], contact with cancer cells [54], or even physiological and genomic stresses [55]. As a consequence of their multiple origins and activation mechanisms, CAFs display different phenotypes and functions in PDAC, also representing an additional source of heterogeneity for this tumor. The function of CAFs as tumor promoters relies on their ability to boost tumor cell proliferation, invasion, and metastasis, as well as their pro-inflammatory and immunosuppressive properties [56]. Furthermore, CAFs have been shown to play a protective role towards cancer cells exposed to radiation or chemotherapy [57]. Despite their well-established role in supporting tumor progression, some studies have observed that CAF depletion results in higher proliferation and migration abilities of tumor cells, thus highlighting a possible role of CAFs as a barrier which is able to limit the spread of PDAC cells [58]. According to these findings, different subtypes of CAFs have been recently identified: myofibroblastic CAFs (myCAF), expressing α-SMA and characterized by periglandular localization, and inflammatory CAFs (iCAF), expressing high levels of pro-inflammatory cytokines and chemokines [59].

#### 2.2.3. Immune Cells

Pancreatic cancer is commonly depicted as an immunologically cold tumor; however, some studies have demonstrated a crucial role of immunological processes in PDAC tumorigenesis [60]. Importantly, inflammation has a tight connection with PDAC development and progression, and chronic pancreatitis is an established risk factor for the development of this cancer [61]. The TME of PDAC is basically immunosuppressive, including regulatory T cells (Treg), myeloid-derived suppressor cells (MDSCs), and tumor-associated macrophages (TAMs) [62].

Tregs (CD4+ and CD25+) are suppressor T cells with the function of preventing autoimmune diseases through the inhibition of effector T cells [63]. In PDAC, they are recruited by tumor cells into the TME, where they contribute to immunosuppression, thus supporting tumor progression; as such, they represent an unfavorable prognostic factor for patients [64]. Tregs exploit their immunosuppressive function through the modulation of several pathways. They can hinder CD8+ T cell activity through the secretion of cytokines and growth factors, such as IL-10 and TGFβ, or by the expression of immune checkpoints (e.g., CTLA-4), leading to effector T cell dysfunction due to the up-regulation of the IDO pathway [65].

MDSCs are a heterogeneous population of cells of myeloid origin which expand during cancer, inflammation, and infection and which play a critical role in suppressing T cell responses [66]. In PDAC patients, a high presence of MDSCs in the peripheral blood may serve as a predictor of chemotherapy failure [67]. In pancreatic cancer, MDSCs are recruited by tumor cells to the TME, mainly as a consequence of granulocyte macrophage colony-stimulating factor (GM-CSF) production [68]. The up-regulation of GM-CSF may be induced by the KRAS^G12D^ mutation, which is detected in almost all cases of PDAC. Once in the TME, MDSCs suppress effector T cells, affecting several different pathways. For instance, they can induce oxidative stress in T cells through the production of ROS, resulting in the impairment of T cell protein translation and leading to a lack of antigen-dependent proliferation [69]. In addition, MDSCs can inhibit T cell proliferation through the depletion of L-arginine from the tumor microenvironment by a STAT3-dependent mechanism. Furthermore, MDSCs have shown an ability to prime and maintain the development of Tregs shaping the tumor stroma [70].

TAMs are M2-like macrophages endowed with immunosuppressive properties which foster tumor progression [71]. They are activated by the IL-4, IL-13, CSF-1, TGF-β, and IL-10 produced by cells in the TME. These secreted factors drive myeloid progenitor cell differentiation into monocytes and macrophages, favoring their recruitment within the microenvironment [72]. The immunosuppressive properties of TAMs are mainly related to the secretion of particular cytokines, chemokines, and enzymes [73]; to their ability to modulate T cell metabolism through the expression of Arg1 in a manner similar to that of MDSCs [74]; and to the ability of these cells to allow for Treg recruitment. Moreover, TAMs sustain PSCs in the deposition of ECM, thus contributing to pancreatic desmoplasia. In particular, TAMs drive PDAC fibrosis through the PI3K pathway and through the production of TGF-β1 and PDGF, which stimulate PSC proliferation and ECM secretion, respectively [75,76]. Consistent with the immunosuppressive functions of TAM, it has been reported that the overall survival of PDAC patients with high M2-like macrophage density is lower when compared to patients with low M2-like macrophage density.

#### 2.2.4. Other Cell Populations

Besides the cell populations mentioned above, endothelial cells and pancreatic cancer stem cells (PCSCs) deserve to be mentioned, even if they are less represented as compared to other cell types within the TME [77]. In PDAC, the low oxygen concentration and the need for nutrients lead to the induction of angiogenesis through the proliferation of endothelial cells from pre-existing vessels. This process, in pancreatic cancer, is regulated by the complex network of different cells composing the TME. For instance, in vitro studies have demonstrated that endothelial cell proliferation is regulated by secreted factors produced by both PSCs and tumor cells [78]. PSCs, upon oxygen restriction, have shown the ability to increase the production of collagen I and vascular endothelial growth factor (VEGF) with pro-angiogenic properties [79]. VEGF is also expressed in tumor cells, endothelial cells, and in TAMs and represents a marker of poor prognosis and high risk of recurrence in pancreatic cancer [80].

Finally, PCSCs are tumor cells that retain the ability to self-renew and to generate heterogeneous cell clones. PCSCs can promote the maintenance of their stemness features by autocrine signaling and influencing the shape of the TME [81]. Stromal cells, in turn, activate paracrine signaling that allows for the preservation of stem cells. In particular, PSCs and TAMs have been reported to promote the self-renewal and functional maintenance of PCSCs [82]. Overall, the main signaling pathways involved in supporting PCSC functions in PDAC are Hedgehog, Notch, and NF-κB, as well as PI3K/AKT and PTEN, all of which are highly de-regulated in this tumor [83].

#### 2.2.5. Non-Cellular Components of the Stroma

The non-cellular portion of the PDAC stroma is mainly represented by a large amount of ECM, composed of fibrillar collagen, fibronectin, and laminin, as well as proteoglycans. ECM-composing molecules are mainly produced by CAFs and, to a lesser extent, PSCs and tumor cells, and they exploit the main function of the physical barrier [66]. Matrix components are able to form a complex network, binding to each other by means of adhesion receptors. A coherent signaling function of the ECM supporting cancer progression has been reported [84].

Collagens comprise 90% of the ECM in PDAC; type I and III are the most abundant, while type IV is less-represented. Notably, type I and III presence increases with the progression from pancreatic intraepithelial neoplasia to PDAC, whilst collagen type IV, together with laminin, divides endothelial cells from other components of the stroma [85]. Collagens shape the mechanical and physical properties of the TME, determining the stiffness of the matrix [86]. Within the TME, collagens are sensed by specific cellular receptors—integrins and discoidin domain receptors—that clusterize upon binding to collagen fibers, leading to the formation of focal adhesions which serve as a connection between the ECM and intracellular proteins. These connections activate multiple signaling pathways involved in the initiation of the EMT, allowing for cell migration [87]. Fibronectin, despite its ability to bind integrins, seems to not be related to EMT; however, it also binds collagens, suggesting an indirect contribution to the promotion of this mechanism [88].

In addition to collagens and fibronectin, hyaluronic acid is highly represented in the PDAC microenvironment. This glycosaminoglycan has the function of maintaining tissue hydration through its water-absorbent properties. However, if present in an excessive amount, it causes an imbalance of interstitial pressure, leading to vessel collapse and hence hampering drug delivery [89].

## 3. Cellular Senescence: An Overview

### 3.1. Cellular Senescence

Senescence was first described as the cessation of cell proliferation of diploid cells caused by telomere shortening [90]. To date, different mechanisms of senescence have been described in several cell types, including fibroblasts [91], epithelial [92], endothelial [93], and mesenchymal stem cells [94], as well as in cancer cells [95]. The main triggers of cellular senescence can be distinguished into two major categories: development-related and stress-related [96]. While the former is involved in embryo development, wound healing, and tissue remodeling [97], damage-induced senescence is involved in pathological manifestations through the generation of a pro-inflammatory microenvironment [98]. Even though the link between aging and senescence is well-defined, these events are not synonymous. In fact, senescence may occur independently of age in response to detrimental signals [99], such as genotoxic insults [100], oncogenic [101] and oxidative stresses [102], epigenetic modifications, chromatin remodeling [103], imbalanced proteostasis [104], or mitochondrial dysfunction [104]. Each of these signals induces a different type of senescence, identified in accordance to the name of the inducing stressor. Replicative senescence, programmed senescence, and stress-induced premature senescence (SIPS) have been previously described. SIPS includes oncogene-induced senescence (OIS), unresolved DNA damage-induced senescence, epigenetically induced senescence, and mitochondrial dysfunction-associated senescence, as well as therapy-induced senescence (TIS) [105]. Despite a lack of unambiguous markers which are able to define senescent cells, it has been well-accepted that senescent cells share some common features regardless of the type of induction [106]. The main characteristics shared by senescent cells are stable growth arrest, resistance to apoptosis, chromatin remodeling, persistent DNA damage response (DDR), increased activity of senescence associated beta-galactosidase (SA-β-gal), and the secretion of multiple pro-inflammatory molecules, which are recognized as hallmarks of the senescence-associated secretory phenotype (SASP) [13,107].

### 3.2. Markers and Molecular Mechanisms of Senescence

As a whole, cellular senescence is described as a response to a stress, either endogenous or exogenous. The first mechanism observed in senescent cells, independent of the origin of the trigger, is cell cycle arrest. The activation of p16 or the p53–p21 axis, converging on the transcriptional inactivation of the retinoblastoma protein (pRb), mainly drives the cell cycle blockade [108]. p16 is a small protein that directly interacts with and inhibits cyclin-dependent kinase (CDK) 4/6. It is frequently considered a specific marker of senescence both in vitro and in vivo [109,110]. p16 expression, at the transcriptional level, is known to be regulated either by epigenetic changes, such as CDKN2A promoter methylation [111], or by transcription factors, such as PPAR-γ, Ets, AP1, and Sp [111]. The increased expression of p16 could be balanced by promoter demethylation or by repressor mechanisms, such as INK4A transcription silence element (ITSE), among others. p16 translation is then modulated through the 5′-UTR region and degraded by N-terminus polyubiquitinylation [112].

p21 is another small protein that inhibits different CDKs. Besides being involved in the trigger of cellular senescence, it is also regulated by p53 in the broader context of DDR, and for this reason, it cannot be considered a specific senescence marker. The p53-independent regulation of p21 is mainly driven by the TGF-β pathway [113]; however, other mechanisms, such as transcriptional repression, control of mRNA stability, phosphorylation, and proteasomal degradation, are known to modulate p21 expression [114]. Moreover, p21 may be indirectly regulated by p16 through the down-regulation of the RNA decay-promoting AUF1 protein [115].

Cell cycle arrest is further supported by the activation of DDR pathways, leading, on the one hand, to p16 and p21 activation and p53 phosphorylation and, on the other hand, to nuclear alterations. Histone H2AX phosphorylation is one of the main detectable alterations of senescent cells. Its activation is induced by double-strand DNA breaks (DSBs) through the binding of ATM kinase to the DNA damage site, and it serves to assemble DNA repair complexes [116,117]. Other histone modifications may occur for the same purpose—for instance, the methylation of histone H3 at lysine 9 (H3K9me3) [118]. The detection of gamma-H2AX nuclear foci and p53 phosphorylation indeed are commonly used as markers of senescence, together with p16 and p21 activation [107].

Additionally, some morphological features can be used to identify senescent cells—increased size and granularity, above all [119]. These morphological parameters may reflect changes in organelle homeostasis and metabolism. Notably, both mitochondria and lysosomes accumulate in senescent cells. Accordingly, increased ROS production, oxidative phosphorylation, and oxygen consumption, as well as lysosomal senescence-associated beta-galactosidase activity, can be assayed to detect senescent cells [120].

### 3.3. SASP

Despite the blockade of cell proliferation, senescent cells remain metabolically active. Accordingly, they secrete a plethora of factors—termed SASP factors—that affect the senescent cell itself as well as non-senescent neighboring cells [121,122]. The exact composition of SASP remains undetermined; however, multiple studies have demonstrated that it is mainly composed of soluble signaling factors, including pro-inflammatory cytokines, chemokines, growth factors, proteases, and matrix metalloproteinases (MMPs), as well as extracellular vesicles [123]. SASP expression is regulated by several pathways, such as nuclear factor κB (NF-κB), CCAAT-enhancer-binding protein β (C/EBPβ), p38 MAPK, serine-protein kinase ATM, and serine/threonine-protein kinase CHK2, and its composition varies according to the type and duration of the trigger [124,125]. In general, DNA damage represents an essential driver of SASP induction [126]. Importantly, SASP factors are not exclusive to senescent cells but may also be expressed by non-senescent ones. CAFs, for example, can secrete factors similar to senescent fibroblasts, being distinguishable from the latter mainly due to their ability to proliferate [16].

The role of SASP in both pathological and physiological processes has been widely identified as dual [99], being potentially beneficial and/or harmful. Many SASP components have immunomodulatory properties [10]. In tumors, for example, interleukin 1 (IL-1), IL-8, and tumor necrosis factor (TNF) are able to recruit immune cells, allowing for the removal of deleterious senescent and neighboring cells in a way that is consistent with the anti-tumor role of senescence [127]. However, the same factors, if chronically expressed, may foster tumor progression, affecting ECM plasticity and vascular permeability [128]. Moreover, SASP factors can also mediate the resistance of senescent cells to immune clearance. Despite the fact that the mechanisms underlying such SASP behaviors have not yet been clearly identified, it is supposed that the accumulation of senescent cells may lead to failed immune clearance and consequent increased SASP production, resulting in boosted senescence induction through the establishment of a feedback loop program [129,130].

Additionally, SASP signaling has been shown to affect the regenerative potential of non-senescent cells [131]. Studies on the regenerative potential of liver cells and keratinocytes in vivo have shown that SASP can affect the regenerative potential through the induction of cell plasticity and stemness [132]. All of these findings have highlighted the complexity and heterogeneity of SASP, thus drawing attention to the necessity of deeply investigating SASP kinetics (Table 1).

## 4. Dual Role of Senescence in PDAC

As described above, four main mutations (*KRAS*, *CDKN2A*, *TP53*, and *SMAD4*) progressively accumulate in pancreatic epithelial cells, driving PDAC tumorigenesis. Importantly, those oncogenes and tumor suppressors are deeply related to senescence, supporting the hypothesis of a substantial involvement of this process in controlling malignant transformations. Senescence in PDAC occurs as an early event [2]. Several studies have reported the detection of senescent cells in early-grade PanIN lesions in both mice and humans. In these lesions, cells stained positive for some of the main senescence markers (e.g., SA-β-gal); showed up-regulation of senescence effectors p16, p53, and p21; and lacked Ki-67 expression [60,139,140]. Coherently with these studies, KRAS activation observed in early PanIN lesions has been shown to trigger OIS, a complex senescence program induced in response to oncogenic signaling [141]. Notably, OIS seems to be further enhanced by the concomitant expression increase of p16 and p53, thus highlighting a protective role of senescence against tumor progression [142]. Despite p16 having been shown to be highly expressed in pancreatic pre-malignancy, frank PDACs displayed inactive p16 in 85% of cases [143]. This evidence paves the way to the hypothesis of senescence bypass in PDAC, allowing for cancer progression [144]. Consistently with this hypothesis, the long-lasting step-wise development and progression of PDAC, as controlled by OIS, can be overcome by the inactivation of p16, p53, and SMAD4—events commonly observed in PDAC [144,145,146]. It is important to note that, while senescence was initially conceived as a stable and irreversible mechanism, its reversibility is now broadly accepted. Several studies have highlighted the inactivation of classical tumor suppressor genes as the most common mechanism for senescence bypass [147,148]. Moreover, senescence bypass in PanIN lesions has been related to the inflammation associated with pancreatitis, which is also one of the major risk factors for PDAC development [61,149]. Guerra et al. have demonstrated that pancreatitis-induced inflammation contributes to PanIN development through the inhibition of OIS. Notably, patients treated with anti-inflammatory therapy showed high senescence levels in low-grade PanIN, suggesting the potential use of such therapy to sustain the protective role of OIS [60]. Other mechanisms involved in senescence bypass are the activation of cyclins and cyclin-dependent kinases [150], the de-regulation of downstream effectors of KRAS (i.e., p38-regulated/activated protein kinase (PRAK) and AKT) [151,152], PTEN loss [153], and the de-regulation of the mechanisms able to sense DNA damage (e.g., gadd45 proteins) [154], as well as epigenetic modifications [155].

Despite the protective role of OIS in PDAC, accumulating evidence has underpinned the role of sustained and prolonged senescence signaling in tumor progression, mainly due to the pro-inflammatory molecules composing the SASP [124,156]. It should be noted that SASP in cancer may play either a pro-tumorigenic or an anti-tumorigenic role. Senescent cells may secrete different factors in variable amounts depending on the type of senescence inducer and on the type of cell undergoing this process. Due to this marked heterogeneity, a common signature to identify SASP-secreting cells is actually lacking. Notwithstanding, a recent study by Jochems et al. has proposed a machine learning-based strategy to detect senescence in in vitro cancer cells [157]. Similarly, Basisty et al. have recently provided the first proteome-based database of SASPs [13]. In general, the main factors secreted by senescent cells include growth factors, such as VEGF, PDGF, HGF, interleukins (IL-1a, IL-6, IL-8, IL-10, IL-13, IL-15), matrix metalloproteinases (MMP3, MMP9), and cytokines/chemokines (CXCL1, CXCL2, CXCL5, CXCL11, CXCL12, CCL2, CCL20). Both tumor and non-tumor cells located in the TME can undergo senescence and are known to secrete different cytokines [158]. In PDAC, as in other inflammatory tumors, the impact of SASP in both tumor and TME evolution remains largely unexplored. Despite this, several SASP molecules, including IL-8, VEGF, and matrix metalloproteinases, have been largely reported in PDAC [159,160]. Furthermore, IL-1α overexpression has been shown to correlate with KRAS mutational status and poor prognosis in PDAC patients [156]. Besides regulating Ras-driven tumorigenesis, IL-1α also triggers NF-κB signaling, which is a pivotal mediator of inflammatory responses [161]. Additionally, increased levels of IL-6 expression in PDAC patient serum were correlated with increased disease burden, reduced performance status of patients, and metastases [162,163]. The presence of IL-6 in the TME has been shown to regulate STAT3 activation both in vitro and in vivo, leading to the proposed role of the IL-6/STAT3 program as a driver of PDAC tumorigenesis [164].

## 5. Senescence in Non-Tumor Cells

**Figure 2:**
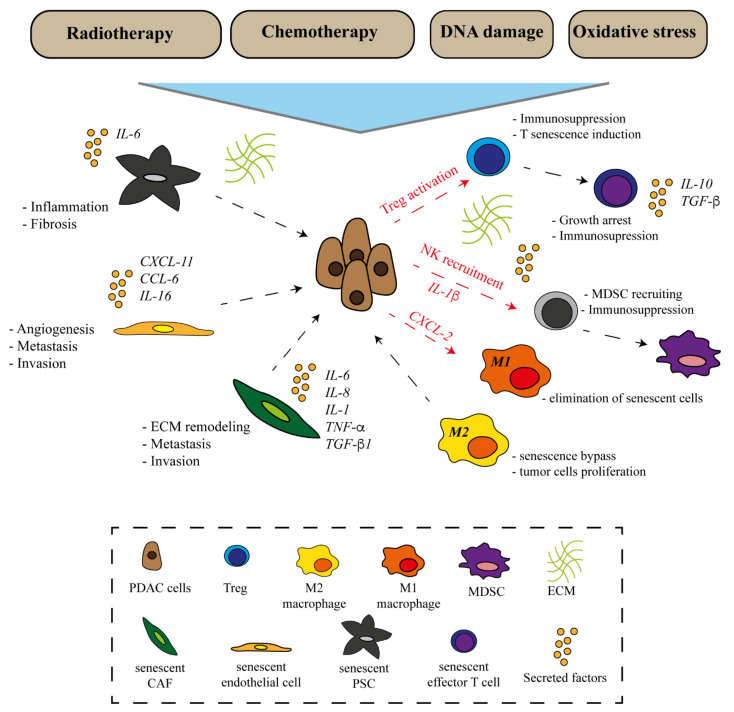
Cross-talk between tumor cells and senescent non-tumor cells in PDAC microenvironment. Abbreviations: PDAC, pancreatic ductal adenocarcinoma; CAF, cancer-associated fibroblast; PSC, pancreatic stellate cell; TAM, tumor-associated macrophage; MDSC, myeloid-derived suppressor cell; Treg, regulatory T cell; ECM, extracellular matrix.

Senescence is a mechanism inducible in both tumor and non-tumor cells [106]. Importantly, one of the main traits of pancreatic cancer is the presence of abundant stroma, a dynamic system that evolves along with the tumor. Studies on the senescence of PDAC stromal cells have shown PSCs to become senescent after sustained exposure to chemotherapeutic drugs (e.g., doxorubicin) or to oxidizing agents (e.g., H_2_O_2_) [165]. Senescent PSCs have shown high CDKN1A expression, together with increased expression of MDM2 and IL-6, while expressing low levels of α-SMA. Importantly, in chronic pancreatitis, the number of senescent PSCs was found to be tightly related to inflammation and fibrosis. In particular, in the regions where the immune infiltrate was more abundant, a high amount of fibrotic- and SAβ-Gal-positive cells was detected, thus suggesting a correlation between PSC activation, inflammation, and senescence in the TME. Subsequent studies on rat PSCs have highlighted the key role of CDKN1A in the maintenance of the senescent phenotype of these cells [165]. Additionally, a study from Shao et al. has further confirmed the role of ROS in inducing senescence in PSCs. The authors claimed that the sqstm1–NRF2 axis, which plays a key role in maintaining the ROS balance, modulates the senescent phenotype of PSCs, thus priming tumor progression [166] (Figure 2).

Compared to PSCs, more studies on senescent CAFs have been conducted in PDAC. CAFs represent a significant portion of the PDAC stroma. Several studies have reported the increase of fibrosis and inflammation in the TME following radiotherapy [167]. Interestingly, a study from Ragunathan et al. showed that a radiation dose higher than 10–12 Gy induced senescence in CAFs together with strong DDR activation [168]. Senescent CAFs, like other senescent cells, present a SASP composed of pro-tumorigenic factors, such as IL-6, IL-8, and osteopontin, which are linked to stroma-mediated therapeutic resistance, besides various cytokines related to radiotherapy-induced fibrosis (i.e., TGF-β1, TNF-α, and IL-1) [169]. In addition, a study from Wang et al. revealed the existence of a senescent CAF population in PDAC endowed with invasion- and metastasis-promoting properties mediated by IL-8 overexpression [170,171]. These CAFs were found to be correlated with reduced survival in patients with early-stage tumors, pinpointing the possible prognostic significance of senescent CAFs. CAFs also represent the main source of ECM production within the TME. It deserves to be mentioned that the ECM, through changes in its composition, may also influence the level of senescent cells in ageing and diseases [172,173].

Endothelial cells are important regulators of the development and function of both blood and lymph vessels. Within the TME, their transformation leads to angiogenesis and vessel alterations, contributing to cancer metastasis. A recent study from Hwang et al. has shown that senescence induced by both doxorubicin and ionizing radiation fosters a pro-tumorigenic SASP. In particular, the production of CXCL11 from endothelial cells was shown to promote the cell proliferation, migration, and invasion of breast cancer cells in vitro [174]. Similarly, Wang et al. have shown that sunitinib-induced senescence stimulates endothelial cells to secrete pro-inflammatory cytokines, thus promoting cancer metastasis and invasion [175]. In contrast with those studies, Ruscetti et al. have demonstrated that the induction of senescence in PDAC cells, through the use of a combination of palbociclib and trametinib, induced vascular remodeling within the tumor, increasing its sensitivity to cytotoxic drugs and promoting T-cell infiltration [176]. This opposing evidence highlights the necessity of improving the knowledge on the effects of senescence in endothelial cells composing the TME.

## 6. Senescence in Immune Cells

Immune cells infiltrating the tumor deserve a separate discussion. It is well-known that different factors trigger senescence in immune cells, among them thymus degeneration, ageing-related decrease in T cell function, and inflammation. Additionally, other immune cell-intrinsic and extrinsic factors, such as those derived from the TME, are also involved [177]. In particular, it has been reported that immune cells may be influenced by SASP. Coherently, immune cells’ senescence could result from a SASP-driven secondary senescence induced by other senescent tumor and non-tumor cells within the TME [178,179]. As for tumor cells, SASP may modulate the immune system with a dual outcome. On one hand, it may promote an immunosuppressive environment, while on the other hand, it may activate the so-called senescence surveillance, which is able to remove senescent cells [180]. Some studies have shown that tumor cells may exploit the SASP to elude immune surveillance. Senescent tumor cells may induce a TGF-β-rich SASP, promoting Treg generation and prompting cell-to-cell senescence transmission. Additionally, a study has shown that Tregs induce senescence in naïve and effector T cells modulating p38, ERK1/2 signaling, and cell cycle-regulatory molecules [181]. Senescent T cells, characterized by a loss of CD27 and CD28 expression and by the secretion of molecules such as IL-10 or TGF-β, have been found to be able to sustain the growth arrest program, supporting the immunosuppressive function of Tregs [182]. Hypoxia within the TME is another factor capable of influencing T cell senescence. A study by Ye et al. has demonstrated that the production of cAMP by tumor cells, induced by the hypoxic environment, is able to trigger senescence in both naïve and effector T cells [182]. Furthermore, tumor cells have also been shown to produce interleukins (e.g., IL-6 and IL-8), which are capable of recruiting myeloid cells that counteract the T cell response [183]. Conversely, senescence surveillance begins when immune cells, such as NKs and macrophages, become activated by the SASP and start to eliminate senescent cells [127]. Chemokines secreted with the SASP, including CCL2, mediate the removal of senescent cells through the NK receptor NKG2D. NK cells are known to be influenced by multiple SASP components [127]. IL-1β, for instance, may modulate NK activity with opposite effects depending on the producing cell type. It has been shown that IL-1β secreted by macrophages increases NK cytotoxicity, while IL-1β produced by tumor cells impairs NK cell development and functions, recruiting MDSCs [184,185]. IL-6 and IL-8, other major components of the SASP, have been found to inhibit the cytotoxic activity of NK cells in different tumors through the down-regulation of perforin and Granzyme B [186]. Furthermore, PGE2 secretion in advanced tumors inhibits NK cell activity, while in the early phases of tumorigenesis, it may promote senescence surveillance. Another cell type involved in immune surveillance is macrophages [178]. Macrophages interact with senescent cells, and this interaction is indispensable for major pathophysiological processes [187]. However, the relation between senescent cells and macrophages is controversial; if on one hand, SASP can lead to senescence surveillance through the recruitment of immune cells [187], on the other, senescent cells recruit macrophages, can induce them to undergo senescence, or can influence their polarization. A recent study by Ogata et al. has reported that senescent cells in the dermis are commonly removed by macrophages in a skin ageing model; however, when those cells accumulate over a certain level (e.g., following oxidative stress, UV exposure, or other stresses), the SASP suppresses the macrophage-related immune clearance of such cells [188]. Additionally, Covarrubias et al., recently demonstrated that the accumulation of senescent cells promotes an inflammatory state associated with CD38 expression and proliferation in M1-like macrophages. This overexpression, promoted by the SASP, leads to enhanced NADase activity which is known to be related to NAD level decline, a common condition in ageing-related diseases [187]. Furthermore, in a PDAC model, OIS-derived SASP factor CXCL1 leads, on one hand, to M1 macrophage recruitment, thus inhibiting carcinogenesis; meanwhile, on the other hand, M2 macrophages are engaged at late stages together with OIS bypass, thus enhancing tumor proliferation [189]. Such evidence, once again, highlights the dual role of SASP, whose effects depend not only on the cell type involved, but also on the stage of tumorigenesis.

## 7. Conclusions and Perspectives

In the early stages of PDAC development, senescence plays a protective role, hindering oncogenic KRAS activation. However, transformed cells along the process of tumorigenesis continuously fight to tackle senescence. Although the mechanisms leading to senescence bypass still remain to be clarified, the acquisition of loss-of-function mutations in tumor suppressor genes, such as TP53, CDKN2A, and SMAD4, leads to the establishment of a highly aggressive tumor [190]. Multiple studies have reported that senescence increases in normal tissues can be connected to tumor progression, thus highlighting a potential role of the senescent stroma in supporting age-related cancers [11]. However, senescence in non-tumor cells is not only related to ageing; DNA damage and oxidative stress caused by systemic cancer therapies are known to trigger senescence in normal cells [55]. Evidence for this has come from patients receiving radio- or chemotherapy showing accelerated biological ageing [185] and from survivors of childhood cancers having an increased risk of developing secondary tumors [191]. For PDAC patients, beyond surgery, the most effective treatment option is represented by systemic chemotherapy, which is administered in all eligible patients, independent of tumor stage, genetic, and histological characteristics [192]. Such wide use of chemotherapy has a valuable effect not only on tumor cells, but also on the cells composing the TME. Although therapy-induced senescence is generally considered a favorable event for patient prognoses, the persistence of senescent cells has been shown to increase the risk of oncogenic transformation and to impair tissue functions. Interestingly, it has been reported that the elimination of senescent cells prevents or mitigates senescence-related diseases [157,177,193]. Strategies to target senescence in cancer have emerged in the pharmacological panorama. In particular, an innovative “one–two punch” approach could pave the way for the use of senotherapeutics in cancer treatment. This approach consists of senescence induction in both tumor and non-tumor cells through the administration of anti-cancer drugs at clinical doses followed by a senotherapeutic drug which selectively eliminates senescent cells. In this way, on one hand, tumor progression, relapse, and drug resistance could be prevented, thus avoiding the detrimental accumulation of senescent cells within the tumor; meanwhile, on the other hand, therapeutic side effects may be alleviated by removing the senescent cells from normal tissue. Despite the great promise of this approach, several issues remain to be addressed to allow for the clinical use of senotherapeutics. First of all, an unambiguous senescence signature allowing senescence targeting in vivo is needed, together with appropriate models representing tumor heterogeneity and 3D architecture exploitable for the development of such drugs.

To conclude, cellular senescence presents a dual function in PDAC which is dependent on the stage of tumorigenesis and the cell type involved. During PDAC establishment, non-tumor cells in normal tissue evolve together with cancer cells, and the cross-talk between tumor cells and TME contribute to the disease outcome. Therefore, the shift from a tumor cell-centric view to a deep knowledge of TME biology is necessary for the development of new and effective therapies against PDAC. Additionally, the progress made in molecular diagnosis regarding circulating proteins, miRNAs, or vesicles may open new scenarios for early diagnosis of PDAC. One of the main advantages of molecular techniques is the non-invasiveness. Tumor-derived SASP, and potentially also proteins derived from senescent non-malignant cells, can be detected and monitored over time in blood, pancreatic juice, or other biological fluids and may be investigated as a useful tool for early diagnosis of PC. In light of this evidence, therapeutic senescence modulation may represent a valuable strategy for the treatment of PDAC.

## Figures and Tables

**Table 1:** Cellular senescence characteristics and biomarkers.

Characteristics	Markers	Ref.
Proliferative arrest	BrDU + Ki-67 - pRB inactivation	Campisi et al. [133]
Persistent DDR activation	p53, p16, p21 up-regulation Lamin B1 down-regulation	Gorgoulis et al. [106] Freund et al. [134]
Heterochromatic foci	gamma-H2AX + DAPI staining	Funayama et al. [135]
Cells flattened and enlarged	Microscopy visible changes	Hernandez-Segura et al. [107]
Apoptosis resistance	Bcl-2; Bcl-XL, Bcl-W expression increase	Childs et al. [136]
Altered metabolism	SA-β-gal + Lipofuscin +	Debacq-Chainaux et al. [137]
Organelle dysfunction	Increased number of mitochondria Morphological changes Increased ROS levels	Gallage et al. [138]
SASP	IL-6; IL-8; MMPs; PAI-1	Coppe et al. [124]

Abbreviations: BrDU, bromodeoxyuridine; pRB, retinoblastoma protein; DAPI, 4′,6-diamidino-2-phenylindole; Bcl-2, B-cell lymphoma 2; Bcl-XL B-cell lymphoma extra-large; ROS, reactive oxygen species; IL-6, interleukin 6; IL-8, interleukin 8; MMPs, matrix metalloproteinases; PAI-1, plasminogen activator inhibitor-1.
